# TXNDC5 is a cervical tumor susceptibility gene that stimulates cell migration, vasculogenic mimicry and angiogenesis by down-regulating SERPINF1 and TRAF1 expression

**DOI:** 10.18632/oncotarget.18857

**Published:** 2017-06-29

**Authors:** Bing Xu, Jian Li, Xiaoxin Liu, Chang Li, Xiaotian Chang

**Affiliations:** ^1^ Medical Research Center of Shandong Provincial Qianfoshan Hospital, Shandong University, Jinan, Shandong, P. R. China; ^2^ Blood Transfusion Department of Shandong Provincial Qianfoshan Hospital, Shandong University, Jinan, Shandong, P. R. China; ^3^ Pathology Department of Tengzhou Central People’s Hospital, Tengzhou, P. R. China

**Keywords:** TXNDC5, cervical tumor, SERPINF1, TRAF1, pathway

## Abstract

TXNDC5 (thioredoxin domain-containing protein 5) catalyzes disulfide bond formation, isomerization and reduction. Studies have reported that TXNDC5 expression is increased in some tumor tissues and that its increased expression can predict a poor prognosis. However, the tumorigenic mechanism has not been well characterized. In this study, we detected a significant association between the rs408014 and rs7771314 SNPs at the TXNDC5 locus and cervical carcinoma using the Taqman genotyping method. We also detected a significantly increased expression of TXNDC5 in cervical tumor tissues using immunohistochemistry and Western blot analysis. Additionally, inhibition of TXNDC5 expression using siRNA prevented tube-like structure formation, an experimental indicator of vasculogenic mimicry and metastasis, in HeLa cervical tumor cells. Inhibiting TXNDC5 expression simultaneously led to the increased expression of SERPINF1 (serpin peptidase inhibitor, clade F) and TRAF1 (TNF receptor-associated factor 1), which have been reported to inhibit angiogenesis and metastasis as well as induce apoptosis. This finding was confirmed in Caski and C-33A cervical tumor cell lines. The ability to form tube-like structures was rescued in HeLa cells simultaneously treated with anti-TXNDC5, SERPINF1 and TRAF1 siRNAs. Furthermore, the inhibition of TXNDC5 expression significantly attenuated endothelial tube formation, a marker of angiogenesis, in human umbilical vein endothelial cells. The present study suggests that TXNDC5 is a susceptibility gene in cervical cancer, and high expression of this gene contributes to abnormal angiogenesis, vasculogenic mimicry and metastasis by down-regulating SERPINF1 and TRAF1 expression.

## INTRODUCTION

Thioredoxin domain-containing protein 5 (TXNDC5, also called ERp46) has a protein disulfide isomerase (PDI) domain that exhibits high sequence similarity to thioredoxin, a catalyst of the rate limiting reactions of disulfide bond formation, isomerization and reduction. This enzyme interacts with many cellular proteins and contributes to the proper folding and the correct formation of disulfide bonds in proteins in the endoplasmic reticulum [1]. TXNDC5 plays an important role in cell physiologic processes, including oxidative stress, cell aging and a wide range of pathologies, such as rheumatoid arthritis, vitiligo, viral infections, diabetes, neurodegenerative disease and cancer [2–14].

TXNDC5 is implicated in cancer progression. Recent studies have indicated that TXNDC5 is overexpressed in tumor tissues from patients with breast invasive ductal carcinoma, cervical squamous cell carcinoma, colorectal adenoma, esophageal squamous cell carcinoma, gastric carcinoma, hepatocellular carcinoma, non-small cell lung carcinoma, ovarian papillary serous carcinoma, prostate cancer and adrenal cell carcinoma [15–23]. Studies also reported that TXNDC5 expression is positively related to tumor cell differentiation, tumor cell invasion and poor survival of patients with certain tumor types [18, 20]. However, little is known about the pathogenic function and the detailed mechanisms of TXNDC5 during tumorigenesis. This study used PCR arrays, including the Cancer PathwayFinder, Signal Transduction, Angiogenesis and TNF Signaling PCR Arrays, to determine the signaling pathway through which TXNDC5 functions during tumorigenesis. The present study also used a three-dimensional culture system with Matrigel to observe the effect of TXNDC5 on the migratory behavior of HeLa cells. Endothelial cells are main component of capillary vessel. Abnormal angiogenesis is a histological hallmark of tumor tissues. TXNDC5 is highly expressed in endothelial cells, where it provides protection from hypoxia-initiated apoptosis [24], but no direct data have indicated that TXNDC5 contributes to abnormal angiogenesis. This study used a Matrigel-based assay to determine the effect of TXNDC5 on the formation of vascular networks by endothelial cells. In a previous study, we used custom-designed Illumina 384-SNP Vera Code microarrays to genotype 96 tag SNPs (single nucleotide polymorphisms) across the TXNDC5 locus to determine the potential association between this gene and tumor occurrence. A case-control analysis revealed a significant association of rs9505298, rs7771314, rs2815128, rs13210097 and rs9392182 SNPs in the TXNDC5 gene with cervical carcinoma, esophageal carcinoma, and liver cancer [23], but the results were not verified in independent cohorts with large numbers of samples using other genotyping method. The current study aimed to confirm this genetic result using Taqman genotyping methods in a new cohort of patients with tumors.

## RESULTS

### Genotyping SNPs located in the TXNDC5 locus

Five SNPs in the TXNDC5 locus, including rs2815128, rs408014, rs4959462, rs7763203 and rs7771314, were genotyped using the Taqman genotyping method in patient cohorts of breast cancer, cervical carcinoma, colorectal cancer, esophageal carcinoma, gastric carcinoma, liver cancer, lung cancer, and rectal carcinoma as well as healthy controls. The allele frequencies and gene frequencies of these SNPs did not deviate from HWE in either the cases or the controls. The case-control analysis exhibited a significant difference in the allele frequency of rs408014 (odds ratio = 1.328862; 95% CI = [1.035893∼1.704689], *p =* 0.025157) between cervical carcinoma patients and controls. The analysis also revealed a significant difference in the allele frequency (odds ratio=0.512561; 95% CI = [0.303152∼0.866624], *p =* 0.011327) and the gene frequency (*p =* 0.033817) of rs7771314 between cervical carcinoma patients and the controls. In addition, the analysis revealed a significant difference in the allele frequency (odds ratio = 0.553230; 95% CI = [0.323325∼0.946612], *p =* 0.028771) of rs2815128 between breast cancer patients and controls. Following a multiple-test correction, these three SNPs still exhibited significant differences with respect to their allelic frequencies and genotypic frequencies between cervical carcinoma patients and controls as well as between breast cancer patients and controls. Significant differences in the allelic or genotypic frequencies of rs4959462 and rs7763203 (*p >* 0.05) were not detected by genotyping among patients with breast cancer, cervical carcinoma, colorectal cancer, esophageal carcinoma, gastric carcinoma, liver cancer, lung cancer or rectal carcinoma and the controls. The above results are presented in Supplementary Table 1.

### Detection of TXNDC5 expression in tumor tissues

**Figure 1:**
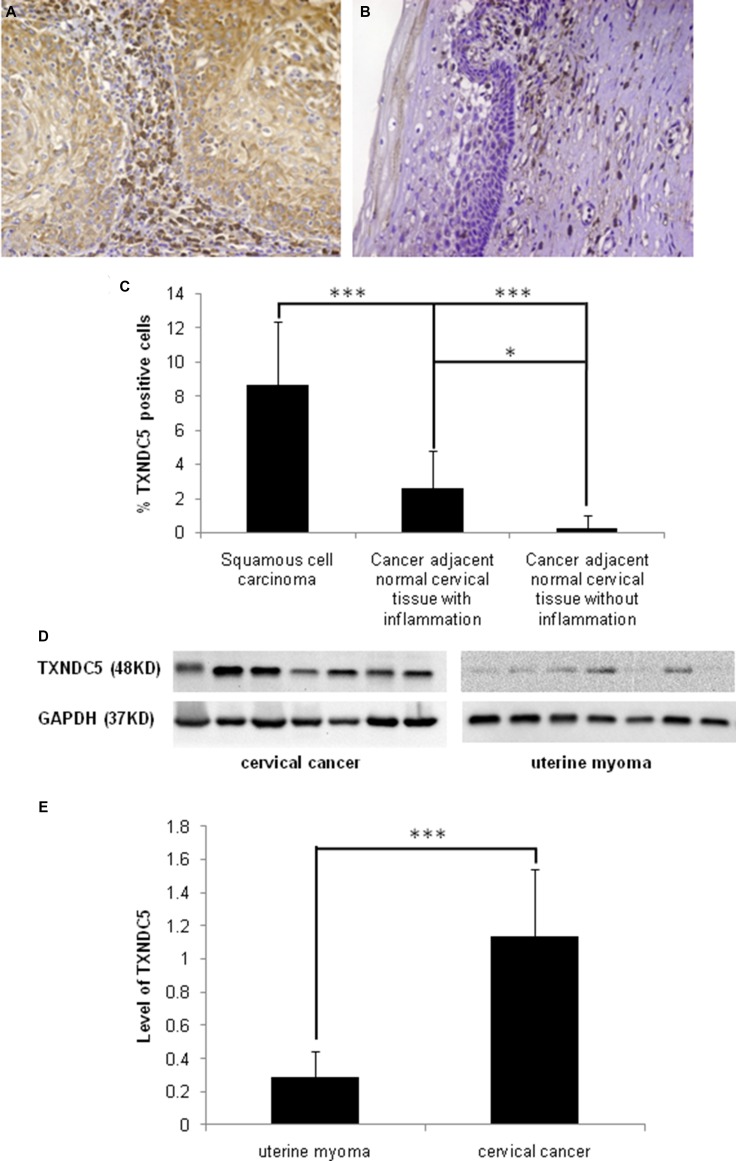
Detection of TXNDC5 expression in cervical carcinoma tissues (**A**) Immunohistochemistry detected significant expression of TXNDC5 in cervical carcinoma tissue. (**B**) TXNDC5 expression was not significantly expressed in normal cervical tissue adjacent to the tumor. Original magnification × 20. (**C**) The immuno-reactive score analysis indicated significantly increased TXNDC5 expression in cervical squamous cell carcinomas compared with the corresponding adjacent normal tissues. (**D**) Western blotting detected a 48-kDa band in tissue extracts from all 7 cervical tumor samples and 5 of 7 uterine myoma samples using an anti-TXNDC5 antibody. (**E**) TXNDC5 expression was normalized to GAPDH expression in each of the tissues. The analysis indicated significantly increased TXNDC5 expression in cervical tumor tissues. *indicates *p* < 0.05, **indicates *p* < 0.01 and ***indicates *p* < 0.001.

Immunohistochemistry was performed to determine TXNDC5 expression in a panel of cervical tumor tissues. TXNDC5 was expressed in all 22 (100%) cervical squamous cell carcinomas. The immunoreactivity was localized to the cytoplasm of the tumor cells. Numerous mesenchymal cells within the tumor tissue also exhibited significant TXNDC5 expression. A portion of mesenchymal cells in close proximity to the tumor cells exhibited very strong TXNDC5 immunoreactivity. TXNDC5 expression was also detected in 11 (68.7%) of 16 tumor-adjacent normal tissue samples with chronic inflammation and 2 (25%) of 8 tumor-adjacent normal tissue samples without inflammation. TXNDC5 immunoreactivity in adjacent normal cervical tissues was observed in a few squamous epithelial cells and some mesenchymal cells, but the density of the positive staining was relatively reduced compared with tumor tissues. The immunohistochemical results are presented in Figure 1A and 1B. Immuno-reactive score analysis was performed, revealing significantly increased TXNDC5 expression in cervical squamous cell carcinomas compared with the corresponding adjacent normal tissues with chronic inflammation (*p =* 0.005) and normal tissues without inflammation (*p =* 0.002). The analytical results are presented in Figure 1C.

Western blot analysis using an anti-TXNDC5 antibody detected a 48-kDa band in the tissue extracts of all 7 cervical tumor samples. This immunosignal was also observed in 5 of 7 uterine myoma samples. Following normalization to GAPDH expression in the tissues, TXNDC5 expression was significantly increased in cervical cancer tissues compared with uterine myoma tissues (*p =* 0.0003). The result is presented in Figure 1D and 1E and indicated significantly increased TXNDC5 expression in the malignant cervical tumor compared with benign tissue.

### Determination of the effect of TXNDC5 on the tube-like structure formation of HeLa cells

**Figure 2:**
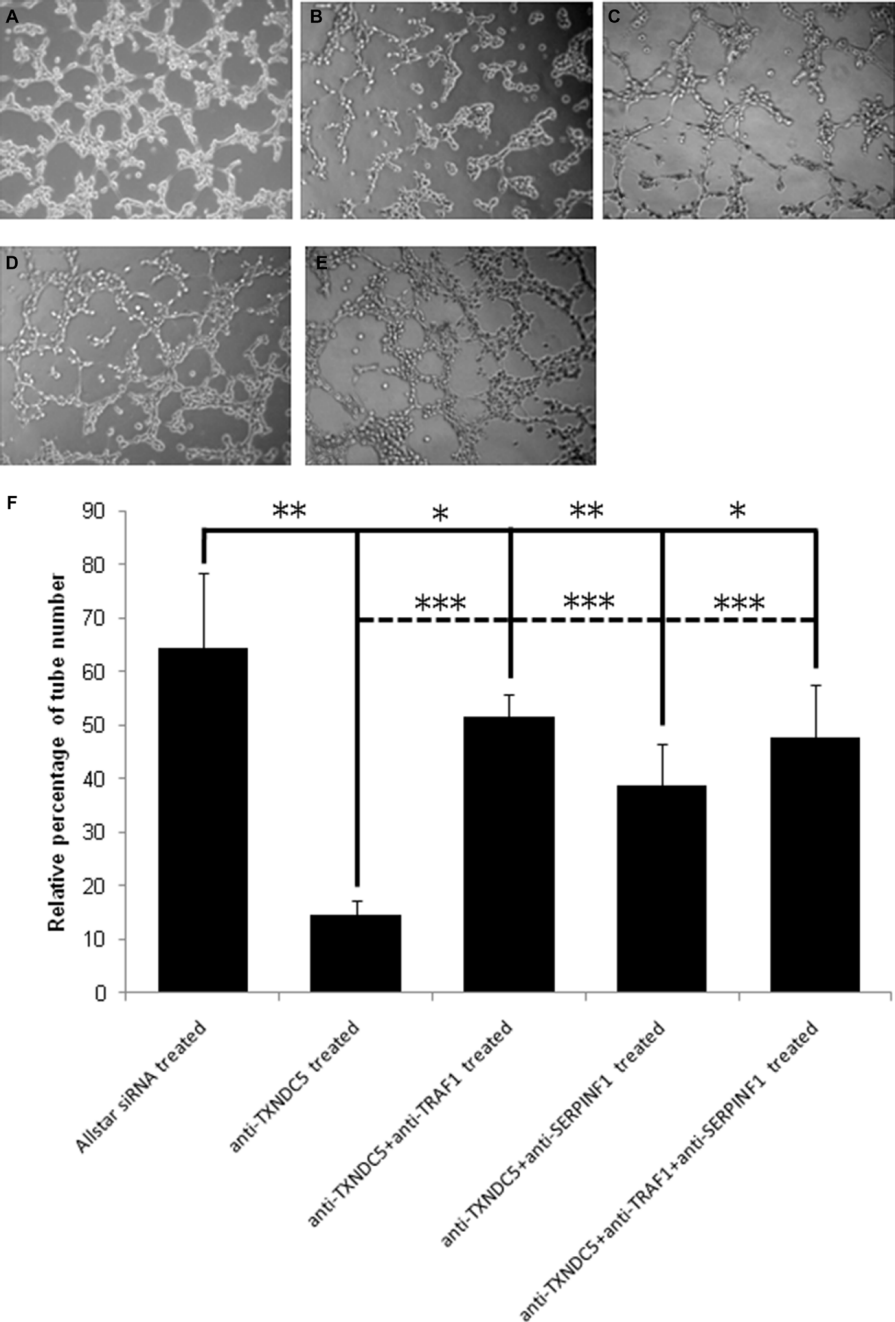
The effect of TXNDC5 on the capability of HeLa cells to form tube-like structures (**A**) HeLa cells treated with AllStars siRNA. (**B**) HeLa cells treated with anti-TXNDC5 siRNA. (**C**) HeLa cells treated with anti-TXNDC5 siRNA and anti- TRAF1 siRNA. (**D**) HeLa cells treated with anti-TXNDC5 siRNA and anti-SERPINF1 siRNA. (**E**) HeLa cells treated with anti-TXNDC5 siRNA, anti- TRAF1 siRNA and anti-SERPINF1 siRNA. (**F**) Quantification of tube-like structures. The number of tubular-like structures was counted using phase contrast microscopy (×10). All test samples were performed in triplicate. *indicates *p* < 0.05, **indicates *p* < 0.01, and ***indicates *p* < 0.001. Full line indicates the difference between AllStars siRNA treatment and other siRNA treatment; dotted line indicates the difference between anti-TXNDC5 siRNA treatment and siRNA mixture treatment.

We treated HeLa cells with anti-TXNDC5 siRNA and observed tube-like structure formation of these cells using a Matrigel assay. Real-time PCR detected a significant 5-fold (*p <* 0.001) decrease in TXNDC5 mRNA expression, and Western blot analyses detected a significant 3-fold (*p =* 0.0017) decrease in the protein level in HeLa cells following treatment with anti-TXNDC5 siRNA. These results are presented in Supplementary Figure 1. HeLa cells subjected to siRNA treatment were cultured in 3D matrix gel in complete medium, and cell migratory behavior was observed by microscopy. As shown in Figure 2, the number of tube-like structures formed by the anti-TXNDC5 siRNA-treated HeLa cells was only one-third the number of tubes formed by HeLa cells treated with AllStars siRNA (*p <* 0.001). These data indicated that the decreased expression of TXNDC5 caused by anti-TXNDC5 siRNA treatment considerably attenuated the migratory ability of HeLa cells and their ability to form tube-like structures on laminin-rich matrix gel.

We simultaneously transfected HeLa cell with anti-TXNDC5 siRNA and anti-SERPINF1 siRNA; anti-TXNDC5 siRNA and TRAF1 siRNA; or anti-TXNDC5 siRNA, anti-SERPINF1 siRNA and TRAF1 siRNA. The tube-like formation ability of the treated cells was observed by microscopy. HeLa cells treated with anti-TXNDC5 and anti-SERPINF1; anti-TXNDC5 and TRAF1 siRNA; or anti-TXNDC5, anti-SERPINF1 and TRAF1 siRNA exhibited significantly increased tube-like structures compared with cells treated with anti-TXNDC5 alone. However, HeLa cells with the siRNA mixture exhibited fewer tube-like structures compared with cells subject to Allstar siRNA treatment. The result is also presented in Figure 2.

### Determination of the tumorigenic pathway of TXNDC5 in HeLa cells

**Figure 3:**
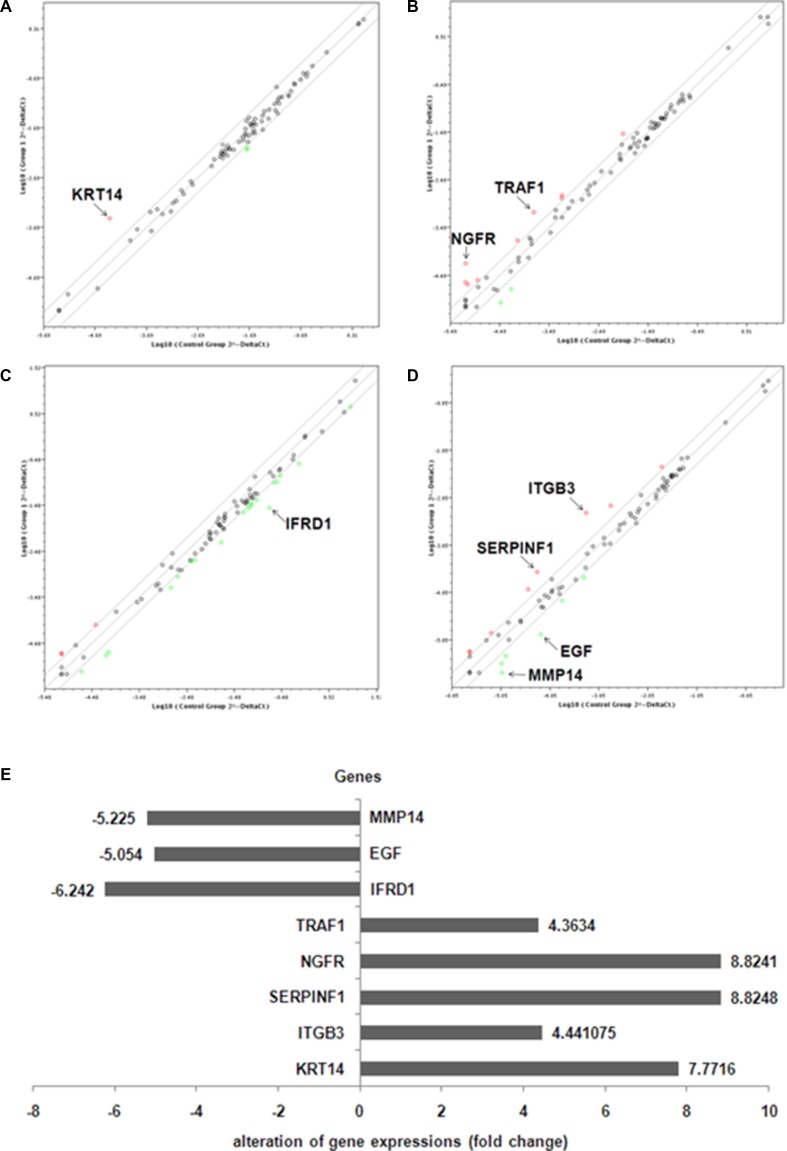
PCR array analysis of relative gene expressions in anti-TXNDC5 siRNA-treated HeLa cells (**A**) Angiogenesis PCR Array, (**B**) Human Cancer PathwayFinderPCR Array, (**C**) Signal Transduction PathwayFinder PCR Array and (**D**) TNF Signaling Pathway PCR Array detected gene expression related to specific signaling pathways. (**E**) The above PCR array results are depicted in one map. The expression levels of MMP14, EGF and IFRD1 were down-regulated, whereas the expression levels of TRAF1, NGFR, SERPINF1, ITGB3 and KRT14 were up-regulated in the siRNA-treated cells.

We further investigated the pathogenic mechanism of TXNDC5 in HeLa cells that originated from a cervical tumor. A series of Qiagen PCR Array analyses were performed to examine changes in gene expression in the siRNA-treated HeLa cells to determine the TXNDC5 signaling pathway involved in tumorigenesis. According to the manufacturer’s instructions, genes that exhibited at least a 4-fold change in expression were considered biologically significant. The Angiogenesis PCR Array detected significantly increased expression of ITGB3 (the integrin beta chain beta 3 or CD61) and SERPINF1 (serpin peptidase inhibitor, clade F, also termed pigment epithelium-derived factor (PEDF)) and significantly decreased expression of EGF (Epidermal growth factor) and MMP14 (matrix metallopeptidase 14 (membrane-inserted)). The Human Cancer PathwayFinder PCR Array detected significantly increased expression of KRT14 (keratin 14). The Signal Transduction PathwayFinder PCR Array detected significantly decreased expression of IFRD1 (Interferon-related developmental regulator 1). The TNF Signaling Pathway PCR Array detected significantly increased expression of NGFR (nerve growth factor receptor) and TRAF1 (TNF receptor-associated factor 1). These results are presented in Figure 3.

**Figure 4:**
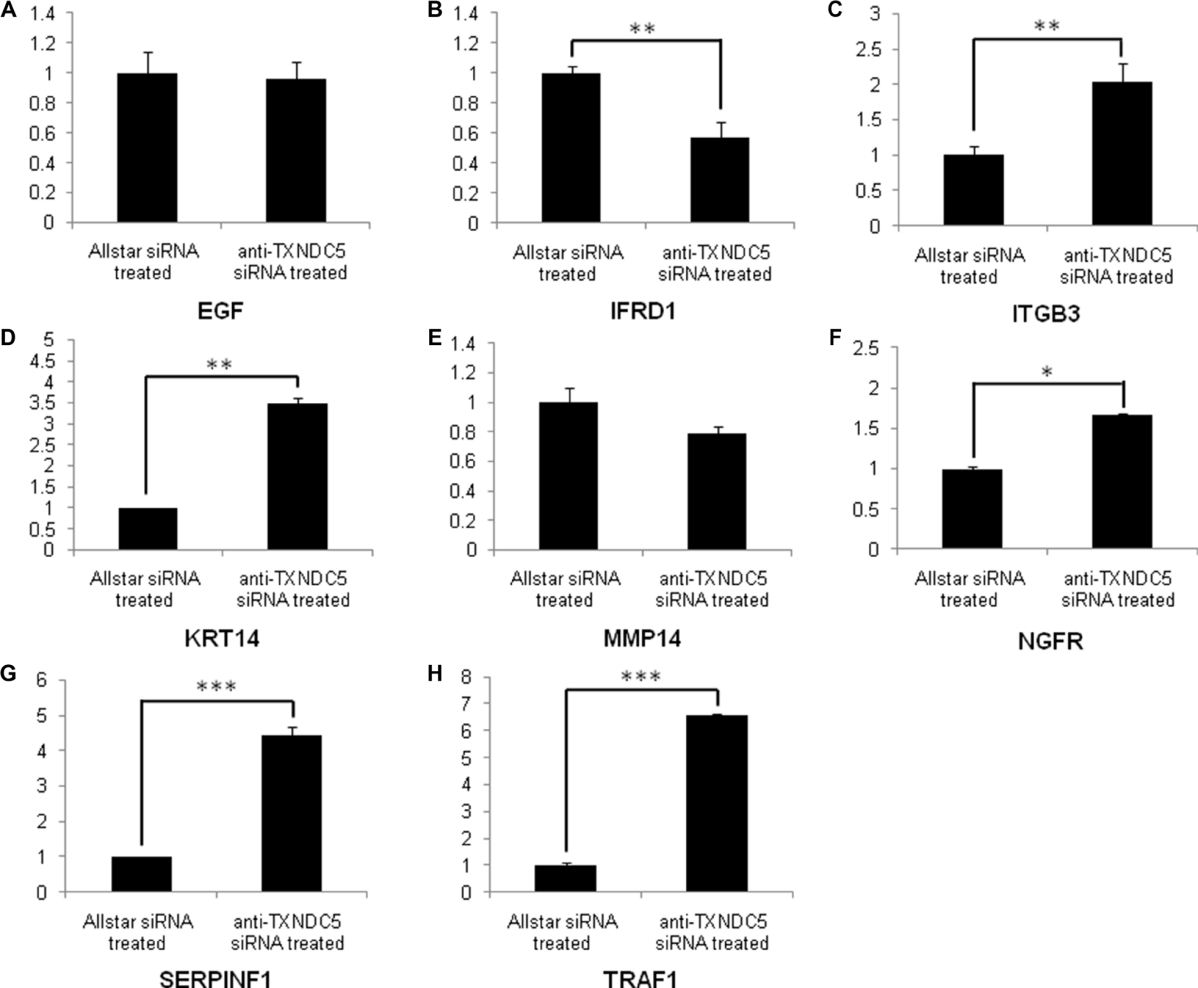
Real-time PCR analysis of EGF, IFRD1, ITGB3, KRT14, MMP14, NGFR, SERPINF1 and TRAF1 expression in HeLa cells treated with anti-TXNDC5 siRNA The transcription levels of the target genes were normalized to GAPDH expression. *indicates *p* < 0.05, **indicates *p* < 0.01, and ***indicates *p* < 0.001.

**Figure 5:**
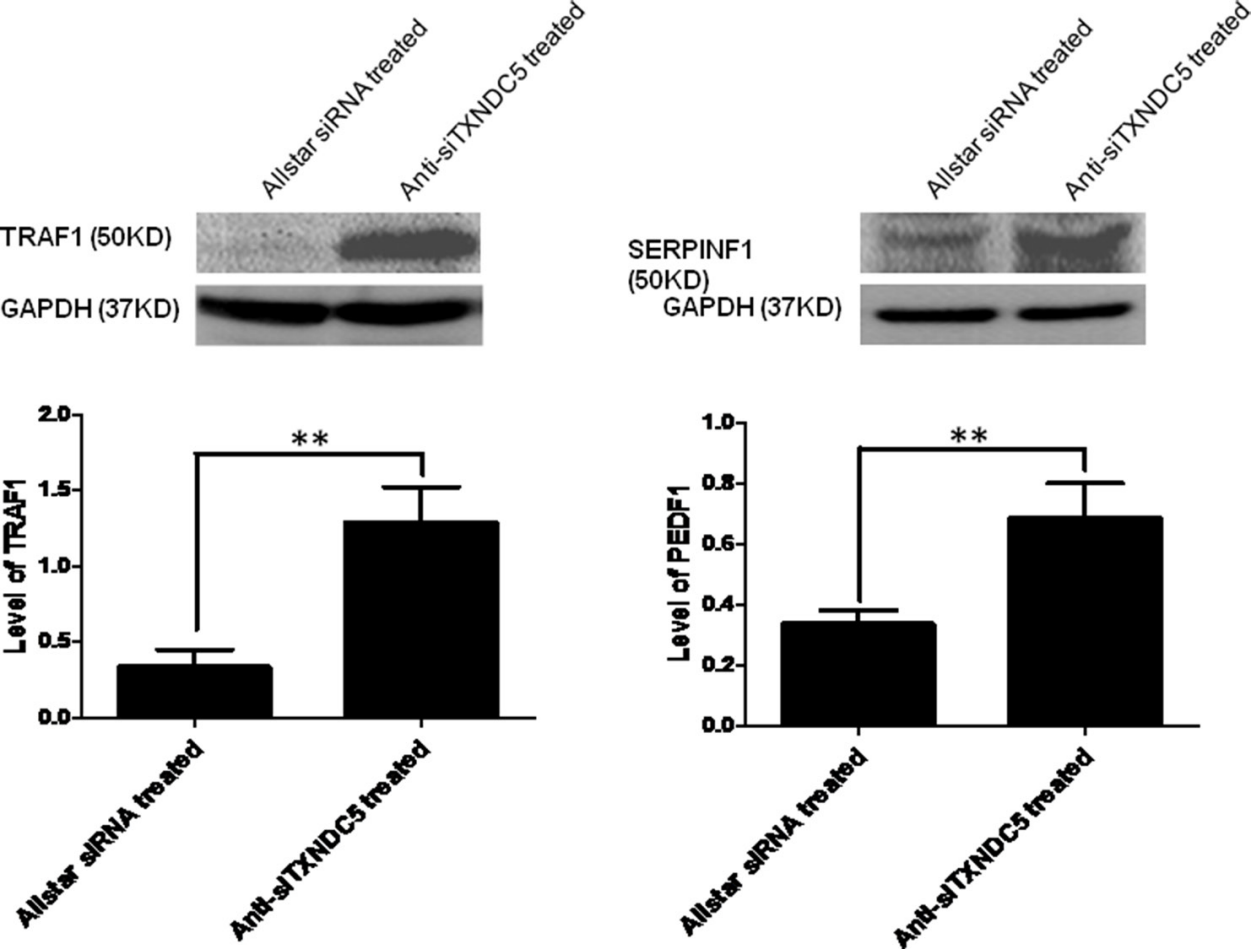
Western blot analysis of SERPINF1 and TRAF1 expression in HeLa cells treated with anti-TXNDC5 siRNA The expression levels of the target genes were normalized to GAPDH expression. **indicates *p* < 0.01.

Real-time PCR was used to verify the expression levels of the target genes described above. Our analysis detected the increased expression of NGFR (*p =* 0.03), ITGB3 (*p =* 0.01), KRT14 (*p <* 0.001), SERPINF1 (*p <* 0.001) and TRAF1 (*p <* 0.001), which were increased 1.67-, 2-, 3.5-, 4.45- and 6.58-fold, respectively, in HeLa cells treated with anti-TXNDC5 siRNA compared with the expression levels of these genes in cells treated with AllStars siRNA. The analysis also detected decreased expression of IFRD1 (*p <* 0.001), which was reduced 1.77-fold in the cells treated with anti-TXNDC5 siRNA. Real-time PCR analysis did not confirm the significantly altered expression of EGF and MMP14. The results presented in Figure 4 verify the greater than 4-fold increase in the transcription of SERPINF1 and TRAF1. Western blot analysis with an anti-TRAF1 antibody detected a band corresponding to a molecular weight of 45 kDa in HeLa cells transfected with the anti-TXNDC5 siRNA. An anti-SERPINF1 antibody also detected a band corresponding to a molecular weight of 47 kDa in treated cells. After the normalization of TRAF1 to GAPDH expression in the cells, TRAF1 exhibited a significant 4-fold increase in expression (*p =* 0.002), whereas SERPINF1 expression differed by 2.5-fold (*p =* 0.004) compared with cells treated with AllStars siRNA. These results are shown in Figure 5. The above results confirmed the increased transcription and translation of SERPINF1 and TRAF1 in HeLa cells treated with anti-TXNDC5 siRNA.

**Figure 6:**
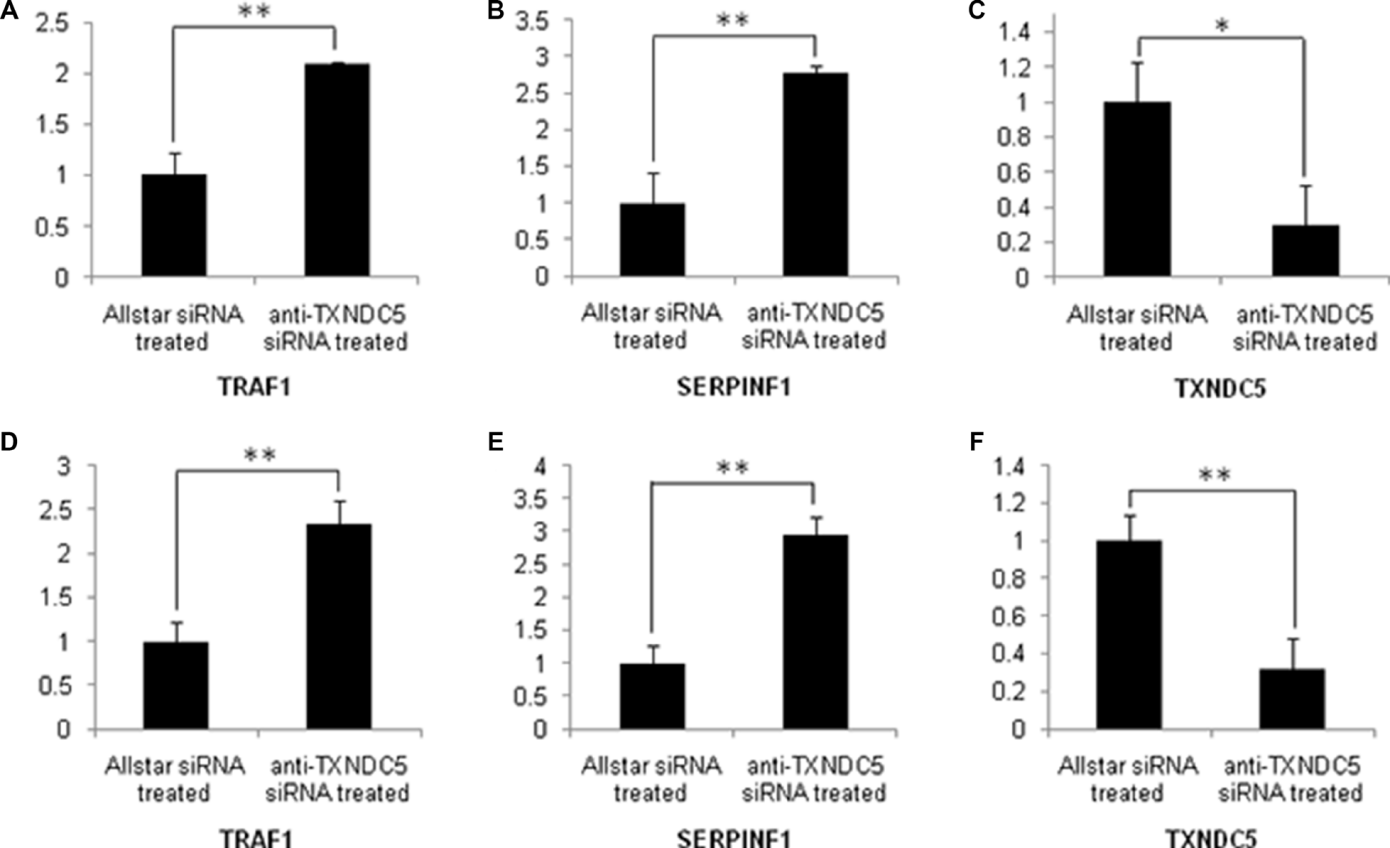
Real-time PCR analysis of TXNDC5, SERPINF1 and TRAF1 expression in Caski and C-33A cells treated with anti-TXNDC5 siRNA The expression levels of the target genes were normalized to GAPDH expression. (**A**–**C**) mRNA expression levels of target genes in sRNA-treated Caski cells. (**D–F**) mRNA expression levels of target genes in siRNA-treated C-33A cells. *indicates *p* < 0.05, and **indicates *p* < 0.01.

**Figure 7:**
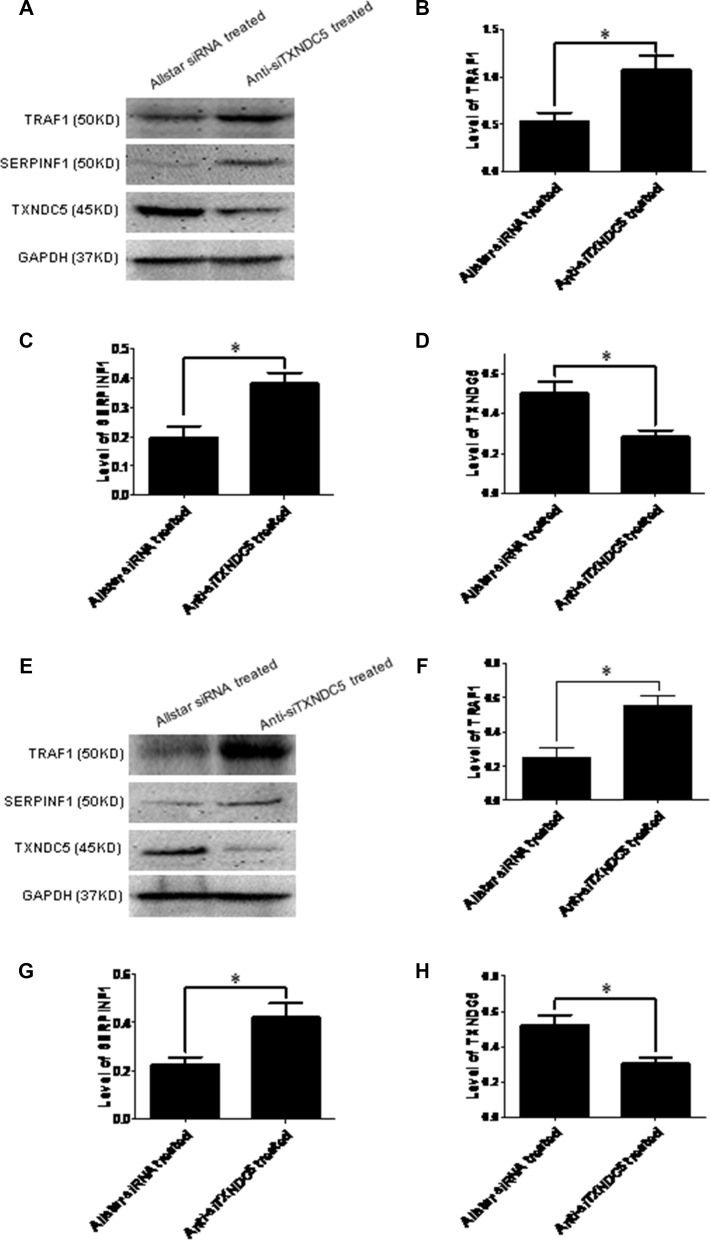
Western blot analysis of SERPINF1 and TRAF1 expression in Caski and C-33A cells treated with anti-TXNDC5 siRNA Western blot analysis of SERPINF1 and TRAF1 expression in Caski and C-33A cells treated with anti-TXNDC5 siRNA. (**A**) Western blot detected SERPINF1, TRAF1, TXNDC5 and GAPDH protein expression in the treated Caski cells. (**B**–**D**) SERPINF1, TRAF1 and TXNDC5 expression in the treated Caski cells was normalized to GAPDH expression. (**E**) Western blot detected SERPINF1, TRAF1, TXNDC5 and GAPDH protein expression in the treated C-33A cells. (**F**–**H**) SERPINF1, TRAF1 and TXNDC5 expression in the treated C-33A cells was normalized to GAPDH expression. *indicates *p* < 0.05.

To confirm the extensive adaptability of down-regulation of TXNDC5 expression to SERPINF1 and TRAF1 expression in the tumorigenic process, Caski and C-33A cervical tumor cells were cultured and transfected with anti-TXNDC5 siRNA. Real-time PCR detected a significantly decreased expression of TXNDC5 mRNA (*p =* 0.001 and *p =* 0.003 in Caski and C-33A cells, respectively), and a significantly increased expression of SERPINF1 (*p =* 0.0019 and *p =* 0.001, respectively) and TRAF1 mRNA (*p =* 0.0193 and *p =* 0.0049, respectively). This result is presented in Figure 6. Western blotting detected significantly decreased TXNDC5 protein expression (*p =* 0.04 and *p =* 0.0317, respectively) and a significantly increased expression of SERPINF1 (*p =* 0.0391 and *p =* 0.0277, respectively) and TRAF1 (*p =* 0.021 and *p =* 0.0376, respectively) protein. This result is shown in Figure 7.

### Determination of the effects of TXNDC5 on angiogenesis in HUVECs

**Figure 8:**
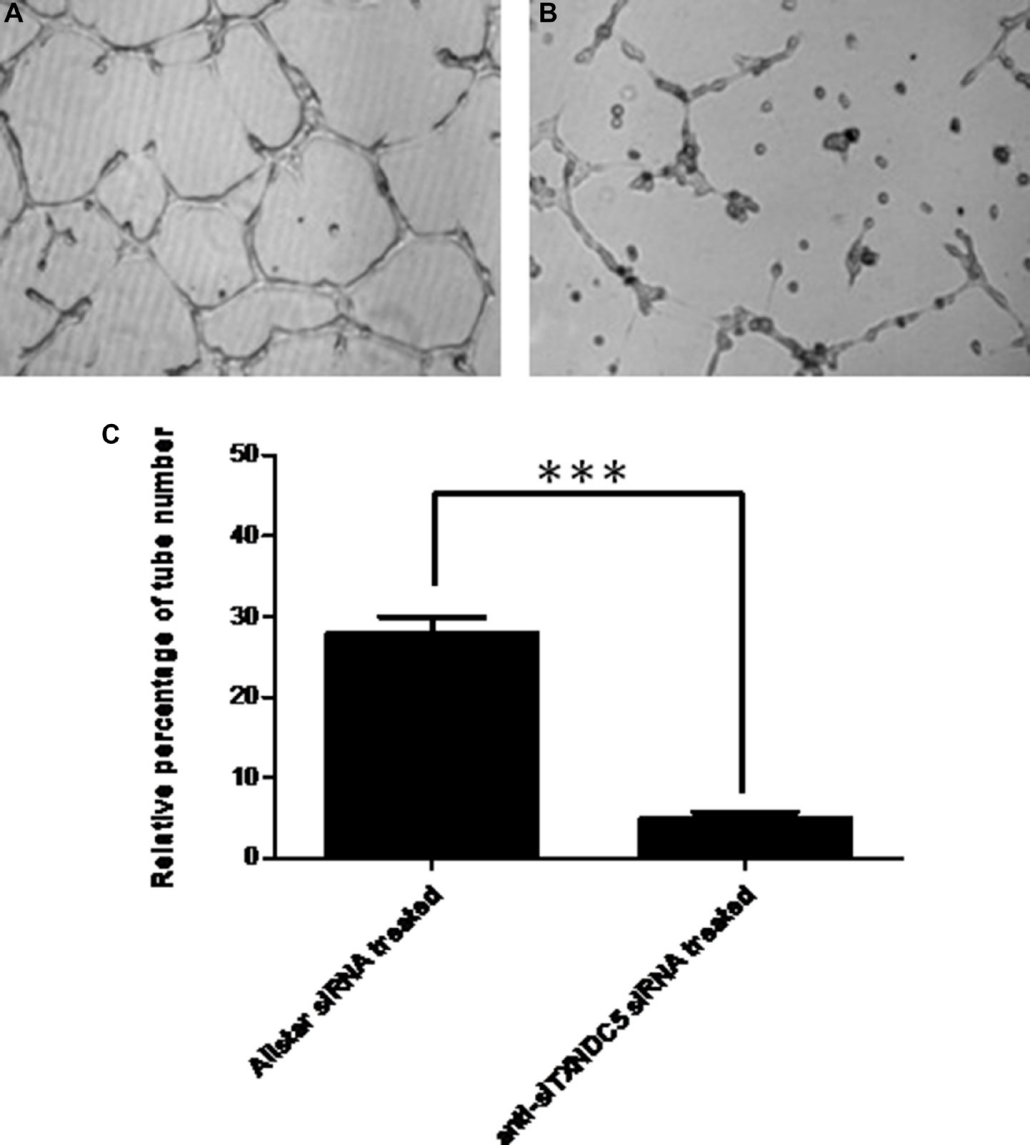
The effect of TXNDC5 on the formation of tube-like structures by HUVECs (**A**) HUVECs treated with AllStars siRNA. (**B**) HUVECs treated with TXNDC5 siRNA. (**C**) Quantification of tube-like structures. The number of tubes was counted using phase contrast microscopy (× 10). All test samples were performed in triplicate. ***indicates *p* < 0.001.

We used a vascular network formation assay with Matrigel to examine whether TXNDC5 expression influences angiogenesis in HUVECs. After treatment with anti-TXNDC5 siRNA, TXNDC5 mRNA and protein expression in HUVECs were significantly decreased by 5- and 3-fold, respectively (*p <* 0.001 and *p =* 0.0025, respectively) as measured by real-time PCR and Western blot analyses. These results are presented in Supplementary Figure 2. As shown in Figure 8, the number of tube-like structures formed by HUVECs was significantly decreased after treatment with anti-TXNDC5 siRNA compared with HUVECs treated with AllStars siRNA (*p <* 0.001). This observation indicates that decreased TXNDC5 expression caused by anti-TXNDC5 siRNA treatment considerably attenuated tube-like structure formation by HUVECs.

## DISCUSSION

In the present study, Taqman genotyping demonstrated a significant association between rs408014 and rs7771314 in the TXNDC5 locus and cervical carcinoma. These two SNPs are located within the intron region of the TXNDC5 gene encoding region. In our previous study, we used custom-designed Illumina 384-SNP VeraCode microarrays to genotype 96 tag SNPs across the TXNDC5 locus. This case-control analysis revealed a significant difference in allele frequencies and genotype frequencies for rs9505298, rs7771314, rs2815128, rs13210097 and rs9392182 among patients with breast cancer, cervical carcinoma, esophageal carcinoma, gastric carcinoma and liver cancer [23]. The results of the current study, which were completed using the Taqman genotyping method, are consistent with results of previous studies and confirms the strong association between rs7771314 and cervical tumor risk. These findings strongly demonstrate that the TXNDC5 gene confers genetic susceptibility to disease risk. This result encouraged us to focus on TXNDC5 expression and its tumorigenic mechanism in cervical carcinoma.

In the current study, we observed extensive immunostaining of TXNDC5 in all cervical squamous cell carcinoma tissue samples. Immuno-reactive score analysis also detected significantly increased TXNDC5 expression in cervical squamous cell carcinomas compared with the corresponding adjacent normal tissues with or without inflammation. Western blot analysis also demonstrated a significant increase in TXNDC5 expression in cervical tumor tissues compared with uterine myoma samples. In addition, some mesenchymal cells in cervical squamous cell carcinoma tissues exhibited significant TXNDC5 expression, and stronger immunostaining was noted compared with tumor cells. Furthermore, the presence of TXNDC5 in mesenchymal cells was detected by immunostaining in 68.7% of normal cervical tissues adjacent to tumors; these tissues also exhibited features of chronic inflammation. A high density of positive staining was also observed in mesenchymal cells in close proximity to the epithelium. However, TXNDC5 expression was not detected in most tumor-adjacent normal tissues in which inflammation was not present. Sullivan *et al.* also reported that TXNDC5 expression is rare in normal tissues but present in the endothelium of tumors and other hypoxic lesions [24], which is in accordance with our results. Wu *et al.* found that the proportion of poorly differentiated gastric adenocarcinomas was significantly increased in tumors with high TXNDC5 expression compared with specimens with low TXNDC5 expression. Lymph node metastasis and the depth of tumor invasion were significantly increased in specimens that exhibited high TXNDC5 expression compared with specimens with low TXNDC5 expression. A survival analysis by the same group revealed that the prognosis of patients who exhibited high TXNDC5 expression was significantly poorer than that of patients who exhibited low TXNDC5 expression [20]. The results from the previous studies and those from the current study confirmed the increased expression of TXNDC5 in cervical tumor tissue. TXNDC5 expression in mesenchymal cells likely plays an important but unknown role in this process.

We previously detected significantly decreased cell proliferation using the CCK-8 kit and by assessing migratory ability of U2OS osteosarcoma cells and HeLa cells following treatment with anti-TXNDC5 siRNA using the Transwell migration assay [23]. Zhang *et al.* detected TXNDC5 expression in HeLa cells, which is in accordance with our results [15]. The silencing of TXNDC5 expression also restrains the growth and proliferation of gastric cancer cells [18]. In the present study using a 3D culture assay, we verified the effect of TXNDC5 on HeLa cell migration. We found that the inhibition of TXNDC5 expression significantly attenuated the capacity of this cervical cancer cell line to form tube-like structures in 3D culture conditions. Aggressive tumor cells can acquire the ability to trans-differentiate into cells with endothelial features, which leads to the formation of vasculogenic networks in a process known as vasculogenic mimicry. Vasculogenic mimicry is associated with increased tumor malignancy and poor clinical outcomes [25, 26]. The *in vitro* tube-like structure formation assay is commonly used to determine the capability of tumor cell lines to form vasculogenic networks. Vasculogenic mimicry has been observed in the ovarian tumor cell line OVCAR-3, the prostate cancer cell lines PC3 and DU-145, and the gastric tumor cell line BGC-823 [27–29]. Wan *et al.* also observed vasculogenic mimicry in HeLa cells [30]. Thus, the previous studies and the present results indicate the essential role of TXNDC5 in tumorigenic activities, including cell proliferation, cell invasion and vasculogenic mimicry in cervical tissues.

The HUVEC-based *in vitro* Matrigel assay is commonly used to analyze neovascularization and angiogenesis. TXNDC5 is highly expressed in endothelial cells and facilitates protein folding in the endoplasmic reticulum [24]. TXNDC5 expression is up-regulated in hypoxic conditions during which it protects endothelial cells from hypoxia-induced cell death [24]. This study investigated the role of TXNDC5 in endothelial angiogenic responses. We found that the inhibition of TXNDC5 expression significantly attenuated the formation of vascular networks by HUVECs. Previous studies as well as our study support the notion that hypoxia-induced up-regulation of TXNDC5 not only protects endothelial cells from hypoxia-induced cell death as previously reported but also stimulates angiogenesis in tumor tissues.

The Human Cancer PathwayFinder PCR Array, the Signal Transduction PCR Array and the Angiogenesis PCR Array were used to identify key novel molecules that regulate the tumorigenic process mediated by TXNDC5 expression in HeLa cells. The increased transcription and translation of SERPINF1 and TRAF1 in anti- TXNDC5 siRNA-treated HeLa cells were detected by PCR array analysis, real-time PCR and Western blotting. To confirm the extensive adaptability of the pathogenic mechanism of TXNDC5, Caski and C-33A cervical tumor cells were cultured and transfected with anti-TXNDC5 siRNA in a manner similar to that described for HeLa cells. Both Caski and C-33A cell lines exhibited significantly increased transcription and translation of SERPINF1 and TRAF1 following treatment with anti-TXNDC5 siRNA. The result is very similar to that observed in HeLa cells with inhibited TXNDC5 expression. This finding strongly suggests that the down-regulation of TXNDC5 on SERPINF1 and TRAF1 expression is a common feature in cervical tumor cells.

TRAF1 is an adapter molecule that regulates the activation of the NF-kappa-B and JNK pathways. TRAF1 and TRAF2 form a heterodimeric complex that interacts with inhibitor of apoptosis proteins (IAPs), and this complex mediates anti-apoptotic signals from TNF receptors [31, 32]. Rajandram *et al.* reported that TRAF1 has both pro-apoptotic and anti-mitotic roles in renal cell carcinoma [33, 34]. Lu *et al.* found that berberine, a natural product that is widely used to treat hyperlipoidemia and intestinal diseases, promoted the up-regulation of TRAF1 levels in treated HeLa cells, demonstrating the involvement of the TRAF1-related death receptor pathway in the process of berberine-induced apoptosis [35]. In addition, Camargo *et al.* found that *ex vivo* tube formation and *in vivo* Matrigel angiogenesis in response to TNF-α were attenuated by anti-TXNDC5 siRNA. TNF-α-stimulated expression of MMP-9 and cathepsin B, two proteases required for angiogenesis, was reduced by TXNDC5 silencing, which blocked the TNF-α-stimulated angiogenic response [36]. The current study found that TXNDC5 regulates TRAF1, a member of the TNF-α mediated signaling pathway. This finding suggests the possibility that TXNDC5 is required for TNF-α-induced angiogenesis by stimulating TRAF1 expression. However, the function of TRAF1 has not been clearly established, and some papers have reported contradictory results. SERPINF1, also termed pigment epithelium-derived factor (PEDF), is a multifunctional secreted protein. SERPINF1 inhibits angiogenesis and metastasis, induces tumor cell apoptosis and differentiation, and activates cellular immunity against cervical cancer [37–40]. SERPINF1 causes regression of immature blood vessels and stimulates the maturation of the vascular microenvironment [41]. Some observations suggest that SERPINF1 can simultaneously inhibit the migration and proliferation induced by vascular endothelial growth factor (VEGF) and further inhibit angiogenesis by interacting with specific cell surface receptors [37]. Thus, the increased expression of SERPINF1 and TRAF1 can explain attenuated vasculogenic mimicry, cell invasion and cell proliferation in anti-TXNDC5 siRNA-treated HeLa cells. The finding also explains the inhibited angiogenesis in HUVECs with reduced TXNDC5 expression.

To confirm the pathway from TXNDC5 to SERPINF1 and TRAF1 that stimulates cell migration and vasculogenic mimicry, we simultaneously transfected HeLa cells with anti-TXNDC5, anti-SERPINF1 and TRAF1 siRNA and observed tube-like structure formation. We found that the treatment of these cells with anti-TXNDC5 and anti-SERPINF1 or anti-TXNDC5 and TRAF1 siRNA considerably rescued the ability to form tube-like structures compared with treatment with anti-TXNDC5 siRNA alone. The result confirms that TXNDC5 activates tube-like formation capability in HeLa cells by mediating SERPINF1 and TRAF1 expression.

The orphan nuclear receptor NR4A1 (nuclear receptor subfamily 4 group A member 1) is overexpressed in various tumors and cancer cell lines. NR4A1 maintains levels of endoplasmic reticulum stress and reactive oxygen species (ROS) in tumor cells to facilitate cell proliferation and survival. Inactivation of NR4A1 decreased TXNDC5 expression, resulting in activation of the ROS/endoplasmic reticulum stress and proapoptotic pathways. Thus, it is possible that NR4A1 exerts pro-oncogenic activity via TXNDC5 [42–44].

Knock down experiments suggest that siRNA cannot be used to simply deduce the phenotype of overexpression. We attempted to collect evidence from HeLa cells that overexpress TXNDC5. However, TXNDC5 exhibits increased expression in cervical tumors, other tumor tissues and various tumor cell lines. Thus, in this study, we could not observe a significant effect of TXNDC5 overexpression on cell proliferation, migratory ability or the formation of tube-like structures by HeLa cells.

In summary, the present study confirmed the significant association between the TXNDC5 gene and cervical carcinoma and demonstrated significantly increased TXNDC5 expression in the tumor tissues. TXNDC5 contributes to the process of vasculogenic mimicry, cell migration and cell proliferation of cervical tumors by down-regulating SERPINF1 and TRAF1 expression. TXNDC5 is also involved in the process of vascular network formation by endothelial cells in the tumors. This finding may be useful for understanding the tumorigenic activities of cervical tumors.

## MATERIALS AND METHODS

### Tissue collection

In the present study, all solid tissue samples and blood samples were collected from patients at the Pathology Department of Tengzhou Central People’s Hospital (Tengzhou, China). Tumor diagnoses were verified by histology, and pathological categorizations were performed according to the World Health Organization (WHO) classification system. All of the included patients signed informed consent, and this study was approved by the Ethics Committee of Shandong Provincial Qianfoshan Hospital and the Ethics Committee of Tengzhou Central People’s Hospital.

### SNP selection and taqman genotyping

The tag SNPs rs2815128, rs408014, rs4959462, rs7763203 and rs7771314 were selected on the basis of linkage disequilibrium patterns that were observed in samples obtained from the Han Chinese population of Beijing, which was genotyped as part of the International HapMap Project. To determine the potential association between these tag SNPs and tumor risk, Taqman was used to genotype patient cohorts with breast cancer (*n =* 326, 326 women, mean age = 47.61), cervical cancer (*n =* 278, 278 women, mean age = 52.75), colon cancer (*n =* 141, 49 women, mean age = 54.16), esophageal cancer (*n =* 280, 40 women, mean age = 61.20), gastric cancer (*n =* 275, 54 women, mean age = 56.87), liver cancer (*n =* 242, 44 women, mean age = 54.3), lung cancer (*n =* 256, 71 women, mean age = 58.77) and rectal carcinoma (*n =* 218, 78 women, mean age = 55.17). Healthy controls were also included in the study (*n =* 285, 71 women, mean age = 38.42). Blood samples were collected and stored in Monovette^®^ tubes containing 3.8% sodium citrate.

Genomic DNA was extracted from whole blood samples with an Omega E-Z 96 Blood DNA Kit (Omega, USA) according to the manufacturer’s protocol. Genotyping assays were run on a ViiA 7 DX Instrument (Life Technologies) and were evaluated according to the manufacturer’s instructions. Each reaction was performed in a total volume of 10 μl using the following amplification protocol: denaturation at 95°C for 10 minutes, followed by 55 cycles of denaturation at 95°C for 15 sec and annealing and extension at 60°C for 1 minute. The genotype of each sample was determined by measuring allele-specific fluorescence using Taqman Genotyper software V1.2 (Life Technologies). Duplicate samples and negative controls were included to verify the accuracy of the genotyping.

Genotyping quality was evaluated using a detailed quality control procedure, which consisted of a > 95% successful call rate, duplicate calling of genotypes, internal positive control samples and Hardy-Weinberg Equilibrium (HWE) testing. SNPs were analyzed for their association with tumor incidence via a comparison of the MAFs (minor allele frequencies) between cases and controls. The dominant and recessive models were considered with respect to the minor allele. Associations between SNPs and tumors were evaluated using odds ratios (ORs) with 95% confidence intervals (CIs). Fisher’s exact test was used for comparisons between categorical variables. P-values less than 0.05 were considered statistically significant. Genotypic association was assessed with SHEsis software [45], whereas the Bonferroni single-step correction was performed with Plink v1.07 (http://pngu.mgh.harvard.edu/purcell/plink/).

### Cell culture and siRNA interference

Cervical cancer-derived HeLa cells were cultured in Dulbecco’s Modified Eagle’s Medium (DMEM) supplemented with 10% fetal calf serum, 50 U/mL penicillin and 50 μg/mL streptomycin in an atmosphere of 5% CO_2_ at 37°C. Human umbilical vein endothelial cells (HUVECs) were purchased from ATCC and cultured in DMEM supplemented with 10% fetal bovine serum in a humidified incubator at 37°C in an atmosphere of 5% CO_2_. SiRNA oligonucleotides targeting the TXNDC5 gene (target sequence: 5′ AGGGCCCTAACTAGAGTTCTA 3′) were designed and synthesized by Qiagen (Germany). The cultured tumor cells or HUVECs were transfected with 20 nM anti-TXNDC5 siRNA using HiPerFect Transfection Reagent (Qiagen) according to the manufacturer’s protocol. The cells were harvested for analysis 48 h after transfection. Negative control cells were transfected with AllStars siRNA, which was provided with the kit, as this sequence cannot suppress the expression of any gene.

To confirm the extensive adaptability of the pathogenic mechanism of TXNDC5, Caski and C-33A cervical tumor cells were cultured and transfected with the anti-TXNDC5 siRNA. TXNDC5, SERPINF1 and TRAF1 expression in these three cell lines was verified by real-time PCR and Western blotting.

### Western blot analysis

Cervical squamous cell carcinoma tissues were collected during surgeries performed in patients with cervical cancer (*n =* 7, 7 females; 44–61 years old, mean 53) or uterine myoma (*n =* 7, 7 females; 36–50 years old, mean 45). One hundred micrograms of each tumor and benign tumor sample was individually homogenized in Cell Lysis Solution (Sigma) and centrifuged at 12,000 × g for 30 min at 4°C. Supernatants were collected after centrifugation, and protein concentrations were determined using a BCA Protein Assay Kit (Pierce). In total, 30 micrograms of protein was loaded and separated by sodium dodecyl sulfate-polyacrylamide gel electrophoresis (SDS-PAGE). Then, the samples were transferred onto a polyvinylidene membrane and probed with an anti-human TXNDC5 antibody (diluted 1:1000). The antibody was prepared by the immunization of a rabbit with a recombinant human TXNDC5 fragment that corresponds to amino acids 198–432 (Abcam). The membranes were subsequently rinsed with wash solution and incubated for 1 h with sheep anti-rabbit IgG conjugated to peroxidase (1:5000; Beyotime Biotech). Following a wash step, the immunosignal was detected using an Enhanced Chemiluminescence (ECL) Plus Kit (Beyotime Biotech). A second membrane was prepared using the same protocol but was probed with an anti-GAPDH antibody (Santa Cruz) to normalize sample loading.

SERPINF1 and TRAF1 expression in the cultured HeLa, Caski and C-33A cells was examined using a protocol similar to the Western blot procedure described above. Commercial antibodies against these two proteins were purchased from Novus and Cell Signaling Technology, respectively. The polyclonal antibody against SERPINF1 was produced by the immunization of rabbits with a His-tagged fusion protein that corresponds to full-length human SERPINF1 expressed in baby hamster kidney cells; the monoclonal antibody against TRAF1 was produced by the immunization of rabbits with a synthetic peptide that corresponds to residues surrounding cysteine 57 of human TRAF1.

### Immunohistochemistry

Tissue array slides were obtained from Alenabio (China). The array slides contained 22 cervical squamous cell carcinoma specimens and 24 adjacent normal cervical tissue samples. Of these samples, 16 samples demonstrated chronic inflammation. Tissue sections were de-paraffinized and rehydrated according to standard procedures. The antibody against TXNDC5 was prepared as described above. The immunoreactivity with dark-yellow color was visualized using the UltraSensitive TM S-P Kit (MaixinBio, China) according to the manufacturer’s instructions. Cell structures were counterstained with hematoxylin.

TXNDC5 expression levels in cervical tumor sections were semi-quantified using the Chiew-Loon Koo’s modified semi-quantitative scoring system, which has been widely accepted and used in other studies [46]. This system considers the staining intensity and extent of the area stained. Every tumor section was scored according to the intensity of the nucleic or cytoplasmic staining (no staining = 0, weak staining = 1, moderate staining = 2, strong staining = 3) and the extent of stained cells (0% = 0, 1–10% = 1, 11–50% = 2, 51–80% = 3, 81–100% = 4; negative means 0% area staining, focally positive means 1–80% area staining, diffusely positive means 81–100% area staining). The final immune-reactive scores were obtained by multiplying the intensity scores with the extent of positivity scores of the stained tissues. The minimum score is 0, and the maximum score is 12.

### PCR array analysis

PCR array is a set of optimized real-time PCR primer assays in a 96-well plate that is used to monitor the expression of genes related to a disease state or pathway. To explore how TXNDC5 is involved in the cervical tumorigenic processes, the Human Cancer PathwayFinder, Signal Transduction, Angiogenesis and TNF Signaling PCR Arrays (Qiagen) were used in the present study. Total RNA was isolated from the anti-TXNDC5 siRNA-treated HeLa cells using TRIzol solution (Invitrogen, USA) according to the manufacturer’s protocol. The experimental RNA samples were reverse-transcribed into first-strand cDNA using an RT^2^ First Strand Kit (Qiagen). The cDNA was mixed with the appropriate RT^2^ SYBR Green Mastermix (Qiagen). This mixture was aliquoted into the wells of the RT^2^ Profiler PCR Array. PCR array analysis was conducted using a ViiA7 DX Instrument (Life Technologies) according to the manufacturer’s instructions. Relative expression levels of the target genes were determined from the data obtained by the real-time cycler and the ∆∆CT method. The raw array data were processed and analyzed by the PCR Array Data Analysis System at http://sabiosciences.com/pcrarraydataanalysis.php as provided by the manufacturer. Fold-changes were calculated and expressed as log-normalized ratios of target gene expression in the anti-TXNDC5 siRNA-treated cells/expression in the AllStars siRNA-treated cells. Genes with at least a 4-fold change in expression were considered biologically significant according to the manufacturer’s recommendation.

### Real-time PCR

Total RNA was extracted from the samples and then reverse-transcribed in a final volume of 10 µl using an RNA PCR Kit (Takara, Japan). Real-time PCR reactions were conducted in a ViiA7 DX Instrument (Life Technologies) according to the manufacturer’s protocol. The reactions were completed in a total volume of 10 µl, which contained the following: 1 µl of cDNA, 5 µl of SYBR Green Real-time PCR Master Mix (TOYOBO, Japan), 1 µl of each primer and 2 µl of H_2_O. PCR amplification cycles were performed under the following conditions: 30 s at 95°C followed by 40 cycles of 15 s each at 95°C and 34 s at 60°C. For each sample, two reactions were performed simultaneously. One reaction was performed to determine the mRNA level of the target gene, whereas the other reaction was performed to determine the level of GAPDH expression. PCR products were confirmed by melt curve analysis. Relative mRNA expression was calculated using the comparative threshold cycle (Ct) method. The relative target gene expression level was normalized to the GAPDH mRNA expression level. The primer sequences are presented in Supplementary Table 2.

### Tumor cell tube-like structure formation assay using matrigel

Matrigel (Corning Life Sciences) was placed dropwise into 24-well culture plates and allowed to polymerize for 1 h at 37°C. HeLa cells treated with anti-TXNDC5 siRNA for 48 h were seeded onto the 3D matrix in complete medium. The number of tube-like structures was counted using phase contrast microscopy (original magnification ×10). Twelve microscopic fields were randomly selected from each well, and the number of tube-like structures per field was counted. All test samples were assayed and counted in triplicate.

To confirm the pathogenic pathway of TXNDC5, we simultaneously transfected HeLa cells with anti-TXNDC5, anti-SERPINF1 or anti-TRAF1 siRNA and observed the tube-like formation ability of the treated cells. The targeting sequence of anti- SERPINF1S siRNA is 5′ CCAGAATTTGACCTTGATA 3′, and the targeting sequence of anti-TRAF1 siRNA is 5′ GAACCCATCTGTCGCTCTT 3′. The cultured HeLa cells were simultaneously transfected with 10 nM anti-TXNDC5 siRNA and 10 nM anti-SERPINF1 or 10 nM anti-TXNDC5 siRNA and anti-TRAF1 siRNA. Cells treated with 20 nM AllStars siRNA were used as a negative control.

### Endothelial tube formation assay using matrigel

Plates (24-well) were coated with 200 μl of Matrigel (Corning Life Sciences) and incubated at 37°C for 1 h to allow the matrix to gel. Human umbilical vein endothelial cells (HUVECs) (5 × 10^4^ cells/well) were seeded on Matrigel-coated plates after treatment with TXNDC5 siRNA or AllStars siRNA for 48 h at 37°C. The number of tubes was counted using phase contrast microscopy (original magnification × 10). Twelve microscopic fields were randomly selected from each well, and the number of tube-like structures per field was counted. All test samples were assayed and counted in triplicate.

## SUPPLEMENTARY MATERIALS FIGURES AND TABLES




