# Supplementary material for: TXNDC5 is a cervical tumor susceptibility gene that stimulates cell migration, vasculogenic mimicry and angiogenesis by down-regulating SERPINF1 and TRAF1 expression
Source: Oncotarget. 2017 Jun 29;8(53):91009–24. doi: 10.18632/oncotarget.18857 (PMC5710901; doi:10.18632/oncotarget.18857)
Supplement: Supplementary file 1 [file oncotarget-08-91009-s001.pdf]

TXNDC5 is a cervical tumor susceptibility gene that stimulates cell migration, vasculogenic mimicry and angiogenesis by down-regulating SERPINF1 and TRAF1 expression

Supplementary Materials

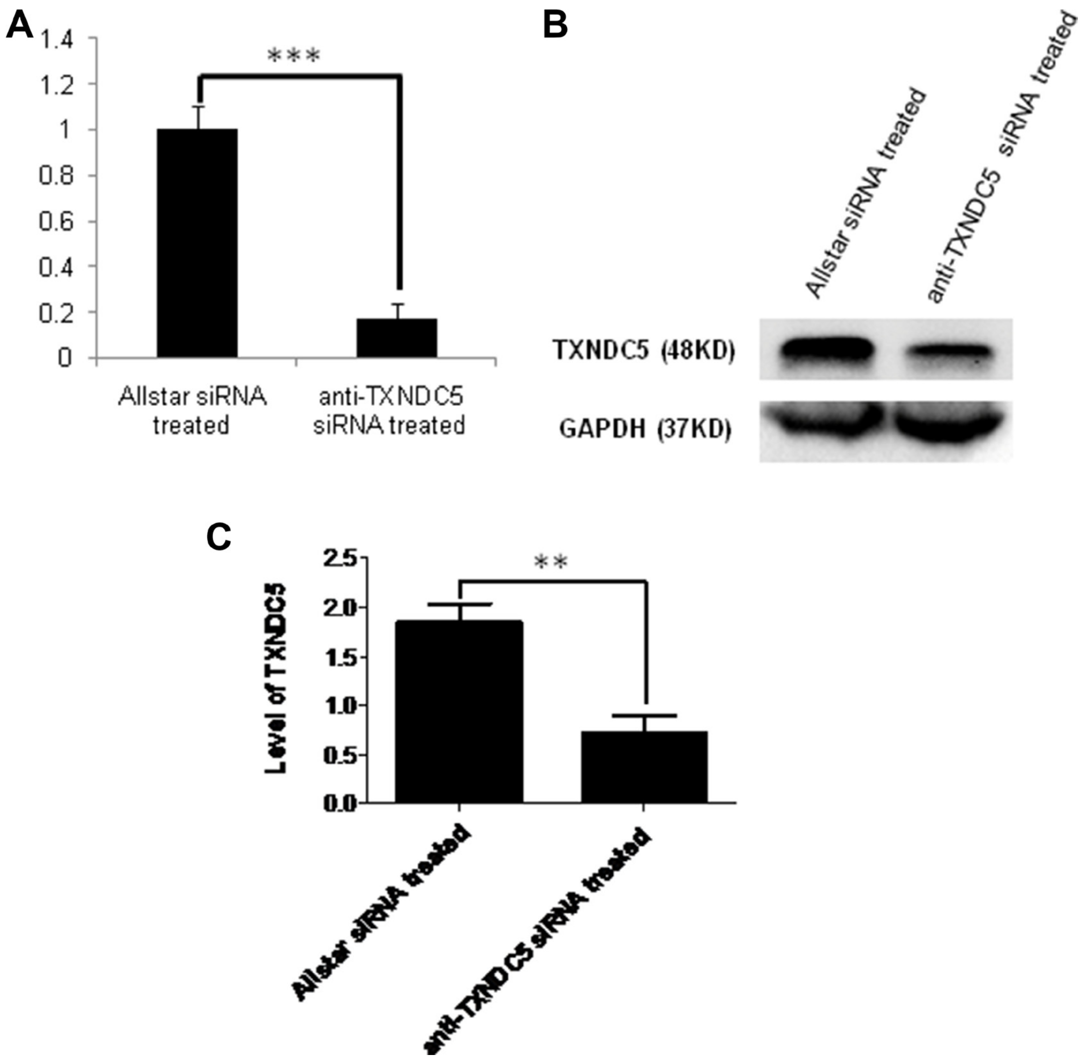

Supplementary Figure 1: TXNDC5 expression in anti-TXNDC5 siRNA-treated HeLa cells. (**A**) TXNDC5 mRNA expression was detected by real-time PCR. (**B**) TXNDC5 protein expression was detected by Western blot analysis. (**C**) TXNDC5 protein expression was normalized to GAPDH expression. Cells treated with AllStars siRNA were used as a negative control. **indicates $p < 0.01$, and *** indicates $p < 0.001$.

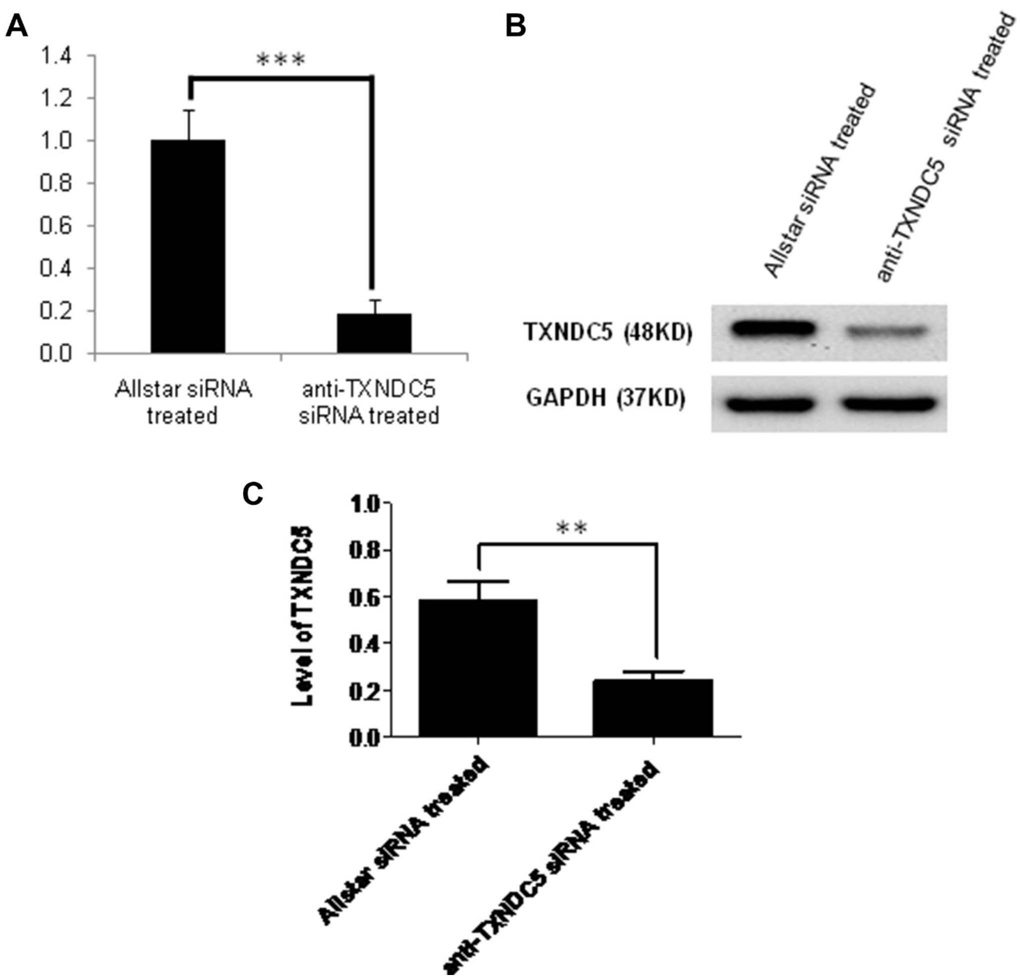

Supplementary Figure 2: TXNDC5 expression in anti-TXNDC5 siRNA-treated HUVECs. (**A**) TXNDC5 mRNA expression was detected by real-time PCR. (**B**) TXNDC5 protein expression was detected by Western blot analysis. (**C**) TXNDC5 protein expression was normalized to GAPDH expression. AllStars siRNA-treated cells were used as a negative control. **indicates $p < 0.01$, ***indicates $p < 0.001$.

Supplementary Table 1: Taqman genotyping result (control *n* = 285). See Supplementary_Table_1

Supplementary Table 2 : Primer sequences of real-time PCR

TXNDC5-Forward:	CTCTGGGCCTTGAACATT
TXNDC5-Reverse:	CCCTCAGTGACTCCAAA
GAPDH-Forward:	CAGAACATCATCCCTGCCTCTAC
GAPDH-Reverse:	TTGAAGTCAGAGGAGACCACCTG
FGF1-Forward:	TTCACAGCCCTGACCGAGAA
FGF1-Reverse:	CGTTGCTACAGTAGAGGAGTTTG
EGF-Forward:	GCTTCAGGACCACAACCATT
EGF-Reverse:	GGCATAAACCATTCCCATCTG
IFRD1-Forward:	GAGTGCGAAGACAAGGCAAG
IFRD1-Reverse:	GCAGCGTTCAATGCTATCAGTT
ITGB3-Forward:	GTGACCTGAAGGAGAATCTGC
ITGB3-Reverse:	CCGGAGTGCAATCCTCTGG
KRT14-Forward:	TGAGCCGCATTCTGAACGAG
KRT14-Reverse:	GATGACTGCGATCCAGAGGA
MMP14-Forward:	CGAGGTGCCCTATGCCTAC
MMP14-Reverse:	CTCGGCAGAGTCAAAGTGG
NGFR-Forward:	TGGCCTACATAGCCTTCAAGA
NGFR-Reverse:	GAGATGCCACTGTCGCTGT
SERPINF1-Forward:	TTCAAAGTCCCCGTGAACAAG
SERPINF1-Reverse:	GAGAGCCCGGTGAATGATGG
TRAF1-Forward:	TGAGAGGGGAGTATGATGCG
TRAF1-Reverse:	GACGCTGAGCTTAGGTCAGG
