# Supplementary material for: TXNDC5 is a cervical tumor susceptibility gene that stimulates cell migration, vasculogenic mimicry and angiogenesis by down-regulating SERPINF1 and TRAF1 expression
Source: Oncotarget. 2017 Jun 29;8(53):91009–24. doi: 10.18632/oncotarget.18857 (PMC5710901; doi:10.18632/oncotarget.18857)
Supplement: Supplementary file 2 [file oncotarget-08-91009-s002.doc]

**Supplementary Table 1. Taqman genotyping result (control n = 285)**

| **rs2815128** | **breast cancer**  **n = 326** | **cervical carcinoma**  **n = 179** | **colon carcinoma**  **n = 125** | **esophageal carcinoma**  **n = 258** | **gastric carcinoma**  **n = 264** | **liver cancer**  **n = 242** | **lung carcinoma**  **n = 179** | **rectal carcinoma**  **n = 218** |
| --- | --- | --- | --- | --- | --- | --- | --- | --- |
| Allele | G T | G T | G T | G T | G T | G T | G T | G T |
| Case (freq) | 25(0.038) 627(0.962) | 23(0.064) 335(0.936) | 12(0.048) 238(0.952) | 27(0.052) 489(0.948) | 37(0.070) 491(0.930) | 26(0.054) 458(0.946) | 16(0.045) 342(0.955) | 27(0.062) 409(0.938) |
| Control (freq) | 32(0.067) 444(0.933) | 32(0.067) 444(0.933) | 32(0.067) 444(0.933) | 32(0.067) 444(0.933) | 32(0.067) 444(0.933) | 32(0.067) 444(0.933) | 32(0.067) 444(0.933) | 32(0.067) 444(0.933) |
| Odds Ratio (%95 CI) | 0.553230 (0.323325~0.946612) | 0.952612 (0.547277~1.658154) | 0.699580 (0.353749~1.383500) | 0.766104 (0.451812~1.299027) | 1.045570 (0.640378~1.707142) | 0.787664 (0.461907~1.343158) | 0.649123 (0.350427~1.202420) | 0.915954 (0.539401~1.555374) |
| Fisher's p value | 0.028771 | 0.863686 | 0.302286 | 0.321546 | 0.858599 | 0.379835 | 0.166712 | 0.745161 |
| Genotype | G/G G/T T/T | G/G G/T T/T | G/G G/T T/T | G/G G/T T/T | G/G G/T T/T | G/G G/T T/T | G/G G/T T/T | G/G G/T T/T |
| Case (freq) | 0(0.000) 25(0.077) 301(0.923) | 0(0.000) 23(0.128) 156(0.872) | 1(0.008) 10(0.080) 114(0.912) | 2(0.008) 23(0.089) 233(0.903) | 1(0.004) 35(0.133) 228(0.864) | 0(0.000) 26(0.107) 216(0.893) | 0(0.000) 16(0.089) 163(0.911) | 0(0.000) 27(0.124) 191(0.876) |
| Control (freq) | 1(0.004) 30(0.126) 207(0.870) | 1(0.004) 30(0.126) 207(0.870) | 1(0.004) 30(0.126) 207(0.870) | 1(0.004) 30(0.126) 207(0.870) | 1(0.004) 30(0.126) 207(0.870) | 1(0.004) 30(0.126) 207(0.870) | 1(0.004) 30(0.126) 207(0.870) | 1(0.004) 30(0.126) 207(0.870) |
| Fisher's p value | 0.072705 | 0.68482 | 0.375893 | 0.36961 | 0.974367 | 0.485837 | 0.334719 | 0.629514 |
| HWE for case (df=1) | 0.471611 | 0.358368 | 0.163492 | 0.104413 | 0.779536 | 0.377218 | 0.531394 | 0.329759 |
| HWE for control (df=1) | 0.937689 | 0.937689 | 0.937689 | 0.937689 | 0.937689 | 0.937689 | 0.937689 | 0.937689 |
| **rs408014** | **breast cancer**  **n = 282** | **cervical carcinoma**  **n = 263** | **colon carcinoma**  **n = 141** | **esophageal carcinoma**  **n = 265** | **gastric carcinoma**  **n = 168** | **liver cancer**  **n = 95** | **lung carcinoma**  **n = 239** | **rectal carcinoma**  **n = 216** |
| Allele | A G | A G | A G | A G | A G | A G | A G | A G |
| Case (freq) | 334(0.592) 230(0.408) | 338(0.643) 188(0.357) | 157(0.557) 125(0.443) | 303(0.572) 227(0.428) | 204(0.607) 132(0.393) | 119(0.626) 71(0.374) | 282(0.590) 196(0.410) | 251(0.581) 181(0.419) |
| Control (freq) | 299(0.575) 221(0.425) | 299(0.575) 221(0.425) | 299(0.575) 221(0.425) | 299(0.575) 221(0.425) | 299(0.575) 221(0.425) | 299(0.575) 221(0.425) | 299(0.575) 221(0.425) | 299(0.575) 221(0.425) |
| Odds Ratio (%95 CI) | 1.073346 (0.842861~1.366858) | 1.328862 (1.035893~1.704689) | 0.928348 (0.693061~1.243512) | 0.986593 (0.772514~1.259997) | 1.142292 (0.863688~1.510768) | 1.238824 (0.880439~1.743091) | 1.063443 (0.826638~1.368085) | 1.024982 (0.791595~1.327179) |
| Fisher's p value | 0.566041 | 0.025157 | 0.618084 | 0.913874 | 0.350932 | 0.218677 | 0.63222 | 0.851528 |
| Genotype | A/A A/G G/G | A/A A/G G/G | A/A A/G G/G | A/A A/G G/G | A/A A/G G/G | A/A A/G G/G | A/A A/G G/G | A/A A/G G/G |
| Case (freq) | 98(0.348) 138(0.489) 46(0.163) | 100(0.380) 138(0.525) 25(0.095) | 48(0.340) 61(0.433) 32(0.227) | 82(0.309) 139(0.525) 44(0.166) | 53(0.315) 98(0.583) 17(0.101) | 37(0.389) 45(0.474) 13(0.137) | 78(0.326) 126(0.527) 35(0.146) | 65(0.301) 121(0.560) 30(0.139) |
| Control (freq) | 79(0.304) 141(0.542) 40(0.154) | 79(0.304) 141(0.542) 40(0.154) | 79(0.304) 141(0.542) 40(0.154) | 79(0.304) 141(0.542) 40(0.154) | 79(0.304) 141(0.542) 40(0.154) | 79(0.304) 141(0.542) 40(0.154) | 79(0.304) 141(0.542) 40(0.154) | 79(0.304) 141(0.542) 40(0.154) |
| Fisher's p value | 0.449373 | 0.051382 | 0.07108 | 0.898949 | 0.289898 | 0.313362 | 0.861073 | 0.881917 |
| HWE for case (df=1) | 0.824928 | 0.021031 | 0.142693 | 0.247283 | 0.003893 | 0.90725 | 0.165748 | 0.026938 |
| HWE for control (df=1) | 0.077299 | 0.077299 | 0.077299 | 0.077299 | 0.077299 | 0.077299 | 0.077299 | 0.077299 |
|  |  |  |  |  |  |  |  |  |
| **rs4959462** | **breast cancer**  **n = 283** | **cervical carcinoma**  **n = 182** | **colon carcinoma**  **n = 134** | **esophageal carcinoma**  **n = 177** | **gastric carcinoma**  **n = 179** | **liver cancer**  **n = 178** | **lung carcinoma**  **n = 256** | **rectal carcinoma**  **n = 217** |
| Allele | C T | C T | C T | C T | C T | C T | C T | C T |
| Case (freq) | 120(0.212) 446(0.788) | 74(0.203) 290(0.797) | 65(0.243) 203(0.757) | 76(0.215) 278(0.785) | 93(0.260) 265(0.740) | 73(0.205) 283(0.795) | 133(0.260) 379(0.740) | 104(0.240) 330(0.760) |
| Control (freq) | 110(0.218) 394(0.782) | 110(0.218) 394(0.782) | 110(0.218) 394(0.782) | 110(0.218) 394(0.782) | 110(0.218) 394(0.782) | 110(0.218) 394(0.782) | 110(0.218) 394(0.782) | 110(0.218) 394(0.782) |
| Odds Ratio (%95 CI) | 0.963718 (0.719629~1.290599) | 0.913981 (0.656155~1.273116) | 1.146888 (0.807968~1.627975) | 0.979202 (0.703822~1.362328) | 0.963718 (0.719629~1.290599) | 0.923932 (0.662263~1.288990) | 1.256944 (0.941184~1.678638) | 1.128815 (0.831765~1.531953) |
| Fisher's p value | 0.80413 | 0.594725 | 0.443015 | 0.900726 | 0.156895 | 0.641401 | 0.120997 | 0.436648 |
| Genotype | C/C C/T T/T | C/C C/T T/T | C/C C/T T/T | C/C C/T T/T | C/C C/T T/T | C/C C/T T/T | C/C C/T T/T | C/C C/T T/T |
| Case (freq) | 14(0.049) 92(0.325) 177(0.625) | 4(0.022) 66(0.363) 112(0.615) | 11(0.082) 43(0.321) 80(0.597) | 6(0.034) 64(0.362) 107(0.605) | 7(0.039) 79(0.441) 93(0.520) | 8(0.045) 57(0.320) 113(0.635) | 16(0.062) 101(0.395) 139(0.543) | 9(0.041) 86(0.396) 122(0.562) |
| Control (freq) | 10(0.040) 90(0.357) 152(0.603) | 10(0.040) 90(0.357) 152(0.603) | 10(0.040) 90(0.357) 152(0.603) | 10(0.040) 90(0.357) 152(0.603) | 10(0.040) 90(0.357) 152(0.603) | 10(0.040) 90(0.357) 152(0.603) | 10(0.040) 90(0.357) 152(0.603) | 10(0.040) 90(0.357) 152(0.603) |
| Fisher's p value | 0.672104 | 0.588156 | 0.198909 | 0.951568 | 0.203975 | 0.720916 | 0.277082 | 0.663393 |
| HWE for case (df=1) | 0.649028 | 0.107058 | 0.142725 | 0.336011 | 0.048388 | 0.812659 | 0.678725 | 0.197324 |
| HWE for control (df=1) | 0.459406 | 0.459406 | 0.459406 | 0.459406 | 0.459406 | 0.459406 | 0.459406 | 0.459406 |
|  |  |  |  |  |  |  |  |  |
| **rs7763203** | **breast cancer**  **n = 281** | **cervical carcinoma**  **n = 177** | **colon carcinoma**  **n = 93** | **esophageal carcinoma**  **n = 280** | **gastric carcinoma**  **n = 275** | **liver cancer**  **n = 190** | **lung carcinoma**  **n = 176** | **rectal carcinoma**  **n = 189** |
| Allele | A T | A T | A T | A T | A T | A T | A T | A T |
| Case (freq) | 543(0.966) 19(0.034) | 341(0.963) 13(0.037) | 178(0.957) 8(0.043) | 545(0.973) 15(0.027) | 536(0.975) 14(0.025) | 374(0.984) 6(0.016) | 340(0.966) 12(0.034) | 363(0.960) 15(0.040) |
| Control (freq) | 532(0.978) 12(0.022) | 532(0.978) 12(0.022) | 532(0.978) 12(0.022) | 532(0.978) 12(0.022) | 532(0.978) 12(0.022) | 532(0.978) 12(0.022) | 532(0.978) 12(0.022) | 532(0.978) 12(0.022) |
| Odds Ratio (%95 CI) | 0.644638 (0.309868~1.341081) | 0.591671 (0.266835~1.311954) | 0.501880 (0.201904~1.247541) | 0.819549 (0.380052~1.767286) | 0.863588 (0.395741~1.884522) | 1.406015 (0.523026~3.779698) | 0.639098 (0.283837~1.439015) | 0.545865 (0.252555~1.179816) |
| Fisher's p value | 0.236675 | 0.191836 | 0.130823 | 0.61123 | 0.712382 | 0.497487 | 0.276014 | 0.118577 |
| Genotype | A/A A/T | A/A A/T | A/A A/T T/T | A/A A/T T/T | A/A A/T | A/A A/T | A/A A/T | A/A A/T |
| Case (freq) | 262(0.932) 19(0.068) | 164(0.927) 13(0.073) | 86(0.925) 6(0.065) 1(0.011) | 266(0.950) 13(0.046) 1(0.004) | 261(0.949) 14(0.051) | 184(0.968) 6(0.032) | 164(0.932) 12(0.068) | 174(0.921) 15(0.079) |
| Control (freq) | 260(0.956) 12(0.044) | 260(0.956) 12(0.044) | 260(0.956) 12(0.044) 0(0.000) | 260(0.956) 12(0.044) 0(0.000) | 260(0.956) 12(0.044) | 260(0.956) 12(0.044) | 260(0.956) 12(0.044) | 260(0.956) 12(0.044) |
| Odds Ratio (%95 CI) | 0.636437 (0.302802~1.337680) | 0.582249 (0.259380~1.307016) |  |  | 0.860440 (0.390524~1.895802) | 1.415385 (0.521720~3.839823) | 0.630769 (0.276787~1.437457) | 0.535385 (0.244688~1.171439) |
| Fisher's p value | 0.229838 | 0.185416 | 0.167031 | 0.608776 | 0.708977 | 0.49316 | 0.269323 | 0.112999 |
| HWE for case (df=1) | 0.557531 | 0.61204 | 0.037043 | 0.067018 | 0.664931 | 0.824997 | 0.639641 | 0.570001 |
| HWE for control (df=1) | 0.709902 | 0.709902 | 0.709902 | 0.709902 | 0.709902 | 0.709902 | 0.709902 | 0.709902 |
| **rs7771314** | **breast cancer**  **n = 188** | **cervical carcinoma**  **n = 278** | **colon carcinoma**  **n = 140** | **esophageal carcinoma**  **n = 183** | **gastric carcinoma**  **n = 185** | **liver cancer**  **n = 181** | **lung carcinoma**  **n = 173** | **rectal carcinoma**  **n = 218** |
| Allele | C T | C T | C T | C T | C T | C T | C T | C T |
| Case (freq) | 29(0.077) 347(0.923) | 23(0.041) 533(0.959) | 22(0.079) 258(0.921) | 21(0.057) 345(0.943) | 29(0.078) 341(0.922) | 24(0.066) 338(0.934) | 18(0.052) 328(0.948) | 24(0.055) 412(0.945) |
| Control (freq) | 41(0.078) 487(0.922) | 41(0.078) 487(0.922) | 41(0.078) 487(0.922) | 41(0.078) 487(0.922) | 41(0.078) 487(0.922) | 41(0.078) 487(0.922) | 41(0.078) 487(0.922) | 41(0.078) 487(0.922) |
| Odds Ratio (%95 CI) | 0.992690 (0.605053~1.628672) | 0.512561 (0.303152~0.866624) | 1.012857 (0.590517~1.737255) | 0.723012 (0.419761~1.245343) | 1.010157 (0.615578~1.657655) | 0.843412 (0.500168~1.422209) | 0.651844 (0.368047~1.154474) | 0.691925 (0.411136~1.164483) |
| Fisher's p value | 0.97683 | 0.011327 | 0.962987 | 0.240722 | 0.968103 | 0.522566 | 0.139787 | 0.163655 |
| Genotype | C/C C/T T/T | C/C C/T T/T | C/C C/T T/T | C/C C/T T/T | C/C C/T T/T | C/C C/T T/T | C/C C/T T/T | C/C C/T T/T |
| Case (freq) | 2(0.011) 25(0.133) 161(0.856) | 0(0.000) 23(0.083) 255(0.917) | 2(0.014) 18(0.129) 120(0.857) | 1(0.005) 19(0.104) 163(0.891) | 1(0.005) 27(0.146) 157(0.849) | 0(0.000) 24(0.133) 157(0.867) | 1(0.006) 16(0.092) 156(0.902) | 0(0.000) 24(0.110) 194(0.890) |
| Control (freq) | 1(0.004) 39(0.148) 224(0.848) | 1(0.004) 39(0.148) 224(0.848) | 1(0.004) 39(0.148) 224(0.848) | 1(0.004) 39(0.148) 224(0.848) | 1(0.004) 39(0.148) 224(0.848) | 1(0.004) 39(0.148) 224(0.848) | 1(0.004) 39(0.148) 224(0.848) | 1(0.004) 39(0.148) 224(0.848) |
| Fisher's p value | 0.620947 | 0.033817 | 0.448554 | 0.387596 | 0.967421 | 0.636337 | 0.227108 | 0.307986 |
| HWE for case (df=1) | 0.366374 | 0.471874 | 0.184966 | 0.586913 | 0.889521 | 0.339479 | 0.672123 | 0.389782 |
| HWE for control (df=1) | 0.611061 | 0.611061 | 0.611061 | 0.611061 | 0.611061 | 0.611061 | 0.611061 | 0.611061 |
|  |  |  |  |  |  |  |  |  |
